# Management of Spontaneous Isolated Mesenteric Artery Dissection: A Systematic Review

**DOI:** 10.1177/14574969211000546

**Published:** 2021-03-16

**Authors:** S. Acosta, F. B. Gonçalves

**Affiliations:** 1 Department of Clinical Sciences, Lund University, Malmö, Sweden; 2 Vascular Center, Department of Cardiothoracic and Vascular Surgery, Skåne University Hospital, Malmö, Sweden; 3 Department of Angiology and Vascular Surgery, Hospital de Santa Marta, Centro Hospitalar Universitário de Lisboa Central and NOVA Medical School, Lisboa, Portugal

**Keywords:** Arterial dissection, mesenteric artery dissection, mesenteric ischemia, computed tomography, conservative therapy, pseudoaneurysm

## Abstract

**Background and Aims:**

There are increasing reports on case series on spontaneous isolated mesenteric artery dissection, that is, dissections of the superior mesenteric artery and celiac artery, mainly due to improved diagnostic capacity of high-resolution computed tomography angiography performed around the clock. A few case–control studies are now available, while randomized controlled trials are awaited.

**Material and Methods:**

The present systematic review based on 97 original studies offers a comprehensive overview on risk factors, management, conservative therapy, morphological modeling of dissection, and prognosis.

**Results and Conclusions:**

Male gender, hypertension, and smoking are risk factors for isolated mesenteric artery dissection, while the frequency of diabetes mellitus is reported to be low. Large aortomesenteric angle has also been considered to be a factor for superior mesenteric artery dissection. The overwhelming majority of patients can be conservatively treated without the need of endovascular or open operations. Conservative therapy consists of blood pressure lowering therapy, analgesics, and initial bowel rest, whereas there is no support for antithrombotic agents. Complete remodeling of the dissection after conservative therapy was found in 43% at mid-term follow-up. One absolute indication for surgery and endovascular stenting of the superior mesenteric artery is development of peritonitis due to bowel infarction, which occurs in 2.1% of superior mesenteric artery dissections and none in celiac artery dissections. The most documented end-organ infarction in celiac artery dissections is splenic infarctions, which occurs in 11.2%, and is a condition that should be treated conservatively. The frequency of ruptured pseudoaneurysm in the superior mesenteric artery and celiac artery dissection is very rare, 0.4%, and none of these patients were in shock at presentation. Endovascular therapy with covered stents should be considered in these patients.

## Introduction

**Fig. 1.**
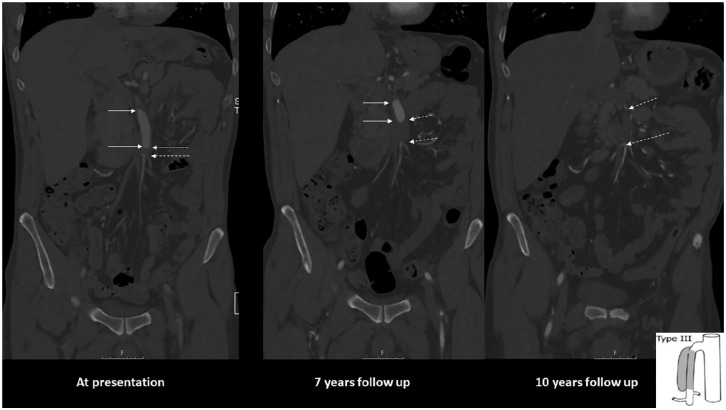
CTA series in the coronal plane of a 48-year-old male patient with symptomatic SMA dissection. He had a history of hypertension and smoking. Onset of acute abdominal pain in his home country. CTA showed suspicion of occlusion of SMA. Explorative laparotomy with lower midline incision found normal small bowels. Appendectomy was performed. He recovered, became rapidly asymptomatic and was prescribed warfarin. Image at presentation (left): there is an entry of dissection 35 mm from the origin of aorta. The length of the dissection is 65 mm and engages the middle and distal SMA. The false lumen is circulated (between arrows). There is a short occlusion of 5 mm distal in the SMA (between dashed arrows). It is a Type III dissection according to Yun et al. (1) (schematic drawing at the lower right corner). Image 7 years later (middle): partial thrombosis of false lumen (between arrows) and progress to 26 mm long thrombotic occlusion of the distal SMA (between dashed arrows). The maximal diameter of the pseudoaneurysm had increased from 11 mm to 16 mm. Image at 10 years of follow-up (right): There is now total occlusion of the main stem of the SMA along the length of the dissection (between dashed arrows). The patient was asymptomatic. The patient died 7 months after this last CT follow-up due to advanced malignancy.

Spontaneous isolated dissection of the superior mesenteric artery (SMA) and the celiac artery (CA) are labeled as isolated mesenteric artery dissections (IMADs). IMADs are increasingly recognized due to widely available high-quality computed tomography angiography (CTA) examination around the clock. For proper diagnosis, imaging has to be performed with intravenous contrast and image acquisition in the arterial phase (Fig. 1). Most patients are diagnosed due to acute abdominal pain at the emergency department. Increased resolution of CTA images has also made it possible to diagnose a percentage of patients with asymptomatic IMAD. Because of the rarity of the diagnosis, high-level evidence is lacking and optimal management and follow-up strategies remain uncertain.

The aim of this systematic review in patients with IMAD is to evaluate risk factors, management, mode of conservative therapy, complete radiological remodeling after conservative therapy, and outcome.

## Methods

**Fig. 2.**
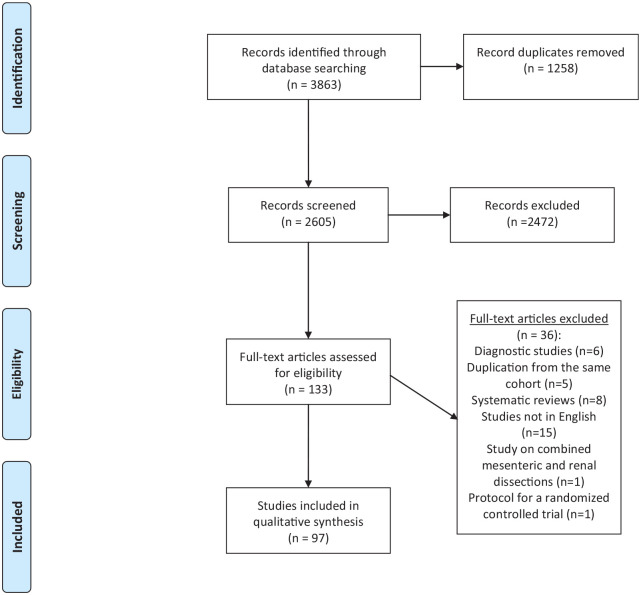
Preferred reporting items for systematic reviews and meta-analyses (PRISMA) flow chart for the review on spontaneous isolated mesenteric artery dissections (IMADs). Source: Moher et al. (98). For more information, visit www.prisma-statement.org.

A systematic literature search strategy of articles on IMAD published from 1 January 1995 to 17 February 2020 was performed using PubMed, Embase, and Cochrane Library databases, for relevant articles published in English. The search was performed with the help of an information specialist and a clinical librarian. In all, 2605 unique abstracts were retrieved after deduplication. The detailed literature search is outlined in Supplemental Appendix, Table 1. Selection of the literature was based on information provided in the title and abstract of the retrieved studies. Only peer-reviewed published literature and studies presenting predefined outcomes were considered. Single case reports with less than five patients, abstracts only, experimental studies and in vitro studies were excluded. Systematic reviews (n = 8), mainly diagnostic studies (n = 6), manuscript in Chinese (n = 14) or Japanese (n = 1) language, protocol for a randomized control study (n = 1) and study on mesenteric and renal dissections without reporting separately for each type of dissection (n = 1) were excluded. If duplication (n = 5) from the same cohort were identified, the latest version was included. Original reports or reports from multi-center collaborations were included. Ninety-seven studies (1–97) were extracted by one author (SA) for this systematic review (Fig. 2). These studies originated from China (n = 31), Korea (n = 24), Japan (n = 21), the United States (n = 13), France (n = 4), Taiwan (n = 1), Brazil (n = 1), and Israel (n = 1). In the study with the largest sample size from the United States (53), 2 out of 77 (2.6%) patients with IMAD were of Asian ethnicity. There was one international multicentre study (the United States, Japan, the Netherlands, France). Ten or more annual original reports were published in the years 2019 (n = 14), 2018 (n = 14), 2017 (n = 12), and 2010 (n = 10).

Data quality was assessed by the European Society of Cardiology system (99): Level of evidence A reflects data derived from multiple clinical trials or meta-analyses; B reflects data derived from a single randomized clinical trial or large non-randomized studies; C consensus of opinion of the experts and/or small studies, retrospective studies, and registries.

## Statistics

Data pooling was performed when feasible from the dataset. Proportions were expressed in percentage with 95% confidence intervals (CIs). Normal distributed variables were expressed as mean with standard deviation (SD). Skewed distributions were expressed as median with interquartile range (IQR). Correlation between two continuous variables was calculated with Pearson correlation.

## Results

### Patients

Ninety-seven studies (evidence level C) with 4239 patients with IMAD were identified. Mean age was 54 years (SD 4.1) in 96 reporting studies. Symptomatic and asymptomatic patients were found in 81% and 19%, respectively, in 93 reporting studies. There were 3408 (80.4%) SMA dissections, 759 (17.9%) CA dissections, 68 concomitant SMA and CA dissections (1.6%), and 4 (0.1%) inferior mesenteric artery dissections.

### Risk Factors

Male gender (88%), hypertension (44%), and smoking (42%) were the most prevalent risk factors for IMAD. Diabetes mellitus was found in a low percentage, 7%, of the patients (Table 1).

**Table 1:** Pooled estimates of risk factors in 4239 patients in 97 studies with IMAD (1–97).

Variable	Studies reporting (N)	Proportion (%)	95% CI (%)
Male gender	95	88	87–89
Hypertension	85	44	43–46
Smoking	71	41	40–43
Diabetes mellitus	58	7	6–8
Hyperlipidemia	49	17	16–19
Cardiac disease, any	30	8	6–10

IMAD: isolated mesenteric artery dissection; CI: confidence interval.

### Morphological Classification of Dissection Based On Ct

An anatomical classification system for describing the extent and severity of the dissection was reported in 52 (53%) studies. The two most common were the Sakamoto (20/52; 38%) and Yun classification (10/52; 19%).

### Potentially Life-Threatening Disease-Related Complications

Ruptured pseudoaneurysm in CA were found in 10 patients in 38 eligible studies and ruptured pseudoaneurysm in SMA in 7 patients in 87 eligible studies. The reported frequency of ruptured pseudoaneurysm was 0.4% (17/4239) among the study patients. None of these patients were in circulatory shock at presentation. Four patients were reported to have been treated conservatively with success. There were 46 (11.2%) splenic infarctions among 412 CA dissections in 11 studies documenting the presence of splenic infarction(s). One splenectomy was performed. All 43 bowel resections were performed in SMA dissections, and the bowel resection rate was 2.1% (43/2092) in 56 studies reporting on SMA dissections only. No bowel resection was reported in studies on CA dissections only.

### Management of Patients

Eighty-two percent of the 4239 patients were treated conservatively. Endovascular procedures were performed in 657 patients: stenting/stent-grafting of true lumen (n = 540), (coil) embolization of false lumen/aneurysm (n = 101), catheter-directed local thrombolysis (n = 18), percutaneous transluminal angioplasty (n = 7), catheter-directed local papaverine infusion at 30 mg/h (n = 4), and in one case the endovascular intervention was unspecified. The sum exceeds 657 since more than one endovascular procedure was performed in the same patient in some cases. Open vascular surgical procedures were performed in 98 patients: bypass from the aorta or iliac segment to the CA or SMA (n = 45) using prosthetic (n = 6), vein (n = 8) or unspecified conduit (n = 31), patch angioplasty (n = 24), thrombectomy (n = 12), interposition graft (n = 8), surgical fenestration (n = 2), resection of dissection and reimplantation of artery (n = 2), aneurysmorrhaphy (n = 2), resection of dissection and direct arterial end-to-end anastomosis (n = 1), resection of dissection and bowel resection (n = 1), and in three cases the surgical repair was unspecified. Some patients underwent multiple arterial surgical reconstructions. Bowel resection was performed in 1.0% (43/4239) of all patients with IMAD (Table 2). The proportion of symptomatic (78.3%; 2374/3031) and asymptomatic (21.7%; 657/3031) patients were reported in 93 (96%) studies.

**Table 2:** Management of 4239 patients in 97 studies with IMAD (1–97).

Variable	Studies reporting (N)	Proportion (%)	95% CI (%)
Conservative	97	82	80–83
Endovascular therapy	97	16	14–17
Open vascular surgery	96	2.3	1.9–2.8
Bowel resection	95	1.0	0.8–1.4

IMAD: isolated mesenteric artery dissection; CI: confidence interval.

### Conservative Management

There were 352 patients in 15 studies reporting on conservative therapy exclusively. The proportion of symptomatic and asymptomatic patients in these 15 studies were 83.2% (293/352) and 16.8% (59/352), respectively. No antithrombotic or anticoagulation therapy was given to 72% of the patients in these 15 studies (Table 3). Among these 15 studies, four reported on symptomatic patients only, and 50% (37/74) received no antithrombotic or anticoagulation therapy.

**Table 3:** Management of 352 patients in 15 studies on IMAD reporting on conservative therapy only (7, 10, 23, 24, 25, 30, 33, 35, 37, 40, 57, 62, 63, 81, 93).

Variable	Studies reporting (N)	Proportion (%)	95% CI (%)
Initial heparin or LMWH	14	18	14–22
Antithrombotic	14	22	18–26
Peroral anticoagulation (warfarin)	12	4	3–7
No antithrombotic or anticoagulation therapy	14	72	68–77

IMAD: isolated mesenteric artery dissection; CI: confidence interval; LMWH: low-molecular-weight heparin.

### Complete Remodeling of Dissection

Complete remodeling (morphological restitution of the artery to its normal condition) at CT follow-up at median time of 22 months (IQR: 13–31) was found in 43% (95% CI: 43–47) of 593 patients in 20 (7, 20, 22, 25, 33, 34, 37, 44, 58, 63, 65, 69, 71, 77, 78, 80, 81, 85, 88, 89) studies on symptomatic IMAD treated conservatively.

### Late Intervention

There were 13 late endovascular (5, 11, 13, 16, 43, 63, 68, 88, 90, 96) and eight open (4, 13, 43, 47, 67, 90, 95) vascular procedures at follow-up (range 1–44 months) reported in 15 studies. The indications for late interventions were occlusive arterial disease (n = 6) and post-dissection aneurysm (n = 9). The indication for late intervention was unspecified in six procedures. The occlusive disease was treated by endovascular stenting due to restenosis after previous stenting (n = 1) and operated plasty (n = 2), and by open surgical bypass (n = 1), thrombectomy plus thrombolysis (n = 1), and thrombectomy plus bowel resection (n = 1). The post-dissection aneurysm was treated by endovascular coil embolization of the false lumen with (n = 4) or without stenting (n = 1), Onyx^®^ embolization of the false lumen (n = 1), and by open resection of aneurysm, aorto-hepatic bypass plus reimplantation of the splenic artery (n = 1) and resection of intima flap plus vein angioplasty (n = 2).

### Mortality

Mortality from IMAD was estimated to be 0.5% or 21 deaths in 3885 patients reported from 93 studies. The number of deaths was correlated to the number of bowel resections performed (r = 0.89; *p* < 0.001). In one report (13), one stent-associated reduction of intestinal perfusion occurred, resulting in intestinal necrosis and death.

## Discussion

The majority of case series stems from East Asian countries. The reason may be multiple. Japan has the largest number of CT scanners among all countries in the Organization for Economic Co-operation and Development (100). South Korea allows CT as part of a general medical check-up (101). China has a large population and most reports of isolated SMA dissection comes from developed regions with high population density (102) and better access to CT scanning. Nevertheless, incidence of CT-verified symptomatic Korean patients with IMAD admitted to the emergency department with abdominal pain was reported to be 0.96%, significantly higher, compared to 0.03% in a comparative Caucasian population (101).

The relation between symptomatic and asymptomatic cases is greatly influenced on methods of retrieval of case series. Series collected by CTA findings from a radiological information system, and not on in-hospital clinical diagnosis based on international classification of diseases codes, will have a larger proportion of asymptomatic cases, which might be chosen to be excluded (20) from the study cohort or not (23). Endovascular therapy has developed and numerous published reports on technical approaches and outcomes exist, frequently excluding patients who were treated conservatively (15). This may result in publication bias toward higher reporting of intervention cases.

The present review confirms that male gender is a risk factor for IMAD. The results also suggest that hypertension and smoking are risk factors, which has been supported by a well-designed case–control study, where symptomatic SMA dissections and controls were matched by clinical presentation, age, gender, and body mass index (84). In addition, increased angulation between the SMA and the distal aorta (> 70°), was found to be associated with symptomatic SMA dissection (84). Large aortomesenteric angle was considered to be an important etiological factor for SMA dissection in another case–control study, which also found that Korean individuals had a larger aortomesenteric angle than Caucasians (101). The low frequency of diabetes mellitus in the present review is unclear. To put these data into perspective, interpretation of data from studies on aortic dissection, a disease with similar patient characteristics and location of primary entry of the dissection on the greater curvature, can be useful. In a nationwide case–control study, individuals with diabetes mellitus had a reduced long-term risk of aortic dissection compared to individuals with no diabetes (103). The reason behind this finding is speculative, but may suggest that glycated cross-links in arterial wall tissue may protect toward arterial dissection.

Emergency CTA has its primary role in the diagnosis of IMAD and evaluation of secondary intestinal ischemic lesions at risk for bowel resection. There is no proof that the morphological classification systems of the dissections described by Sakamoto or Yun dictates clinical decision-making. When there is a clinical indication to operate, CTA-reconstructed images of the dissection may be useful in guiding mode of intestinal revascularization, endovascular, or open vascular surgery. Intestinal revascularization should preferably be performed prior to any bowel resection at the hybrid operation room (104). However, the overwhelmingly majority of patients in the present review were treated conservatively without the need of endovascular or open operations. In fact, there appears to be very few indications for intervention in IMAD patients. One indication is development of peritonitis due to bowel infarction, which on the other hand, will develop in a very small percentage. The need for bowel resection was very low at only 1.0%, and was only necessary in SMA dissections, whereas there were no bowel resections performed in CA dissections. The present review showed clearly that the need for bowel resection was correlated to death in IMAD. The most-documented end-organ infarction in CA dissections was splenic infarction, a condition that should be treated conservatively (23, 54, 56). The frequency of ruptured pseudoaneurysm in SMA and CA dissections was rare. This apparently life-threatening condition was never associated with shock in the reported cases and four patients with ruptured CA dissections (23, 37) out of the 17 documented cases were even treated conservatively without operation. Hence, ruptured pseudoaneurysm as a complication to IMAD based on a CTA finding but not with clinical signs of bleeding may potentially be treated conservatively under strict vigilance. On the contrary, since none of the 97 studies in the review reported patients diagnosed at autopsy, ex-hospital deaths due to intra-abdominal exsanguination cannot be excluded.

Pursuing the endovascular treatment option may cause harm. In an honest and courageous report, there were endovascular technical failures in a large proportion of patients (13), including failure of cannulation of the true lumina, stent thrombosis, and stent misplacements into false lumen. Most importantly, one stent-associated reduction of intestinal perfusion occurred, resulting in intestinal necrosis and death. All five patients undergoing failed endovascular attempts without having a stent placed in the SMA had uneventful outcomes after subsequent conservative therapy, which suggests that there was no indication of endovascular therapy in the first place. The often-stated indications for stenting such as large dissecting aneurysm and/or persistence or aggravation of symptoms (105) during a short period of time, remain speculative and are not supported by current evidence. The CTA features of the large dissecting aneurysm seen at various time points in the case presented in Fig. 1 was indeed worrisome, but no endovascular surgeon at our highly endovascular-oriented center was throughout the long follow-up time interested in planning for a endovascular stent graft procedure covering numerous important arterial branches of the SMA potentially resulting in life-threatening intestinal ischemia in this asymptomatic patient.

The mode of conservative therapy generally consisted of blood pressure lowering therapy, analgesics, and initial bowel rest. However, the need and type of antithrombotic therapy in these patients has been a matter of debate. The majority of patients, 74%, in this review were not treated with any specific antithrombotic or anticoagulation therapy. Since IMAD is not an atherosclerotic disease (106), there is no evidence of beneficial effect of antithrombotic therapy (107). A recent meta-analysis did not recommend the use of additional antithrombotic agents for either symptomatic or asymptomatic SMA dissection, unless further evidence shows any beneficial effect (108).

Endovascular therapy with stenting is inherently associated with increased proportion of complete remodeling of the dissection compared to conservative therapy (65). However, stented patients may need life-long antiplatelet agent to prevent stent thrombosis (65), and some patients will inevitably develop in-stent restenosis (21). Instead, conservative management of IMAD without any antithrombotic medication is a fairly good option, since the chances of complete remodeling after conservative therapy was 43% after a median CTA follow-up time of 22 months in the present review. In addition, late interventions were rarely performed, only 21 documented late interventions in a total of 4239 patients. However, long-term follow-up is generally not reported and late aneurysmal degeneration with or without mesenteric artery occlusion may develop. On the contrary, morphologic changes of the IMAD appears mainly to occur within the first year after onset of disease (25), and serial CT follow-up thereafter does not seem to be appropriate due to harmful exposure of cumulative radiation and iodine contrast to these middle-aged individuals. If imaging follow-up is considered in selected patients, the combination of color duplex ultrasound and contrast-enhanced ultrasound may be a viable and preferred alternative for surveillance (83). In asymptomatic IMAD patients, however, it is very uncertain whether imaging follow-up should be performed at all.

In summary, there are now numerous case series on IMAD with focus on risk factors, diagnosis, treatment, and prognosis. While some retrospective case–control studies (41, 84, 101) have been published lately, more prospective studies with high-quality data and randomized trials in this area are awaited.

## Conclusion

Conservative management is the main treatment option in IMAD patients. There is almost never an indication for stenting in CA dissection. The only absolute indication for endovascular or open vascular surgery is development of severe intestinal ischemia and peritonitis in symptomatic SMA dissection. There is no evidence to support the use of antithrombotic therapy for IMAD patients, unless they have undergone stenting.

## Supplemental Material

sj-pdf-1-sjs-10.1177_14574969211000546 – Supplemental material for Management of Spontaneous Isolated Mesenteric Artery Dissection: A Systematic Review Click here for additional data file. Supplemental material, sj-pdf-1-sjs-10.1177_14574969211000546 for Management of Spontaneous Isolated Mesenteric Artery Dissection: A Systematic Review by S. Acosta and F. B. Gonçalves in Scandinavian Journal of Surgery
