# Supplementary material for: Management of Spontaneous Isolated Mesenteric Artery Dissection: A Systematic Review
Source: Scand J Surg. 2021 Mar 16;110(2):130–8. doi: 10.1177/14574969211000546 (PMC8258720; doi:10.1177/14574969211000546)
Supplement: sj-pdf-1-sjs-10.1177_14574969211000546 – Supplemental material for Management of Spontaneous Isolated Mesenteric Artery Dissection: A Systematic Review [file sj-pdf-1-sjs-10.1177_14574969211000546.pdf]

Literature search: Mesenteric artery dissection

PubMed, 2020-02-17

#1 ("Mesenteric Artery, Superior"[Mesh] OR "superior mesenteric artery" OR "arteria mesenterica superior" OR "mesenteric artery" OR "mesenteric arteries" OR "coeliac trunk" OR "truncus coeliacus" OR "visceral artery" OR "visceral arteries" OR "Celiac Artery"[Mesh] OR "celiac artery" OR “celiac arteries”) 28,385

#2 #1 AND (dissection OR dissection[MeSH Terms]) 1,599

#3 Limit to publication date 1995-01-01 to 2020-02-17 1,461

Embase, 2020-02-17

#1. 'superior mesenteric artery'/exp OR 'superior mesenteric artery' OR 'arteria mesenterica superior'/exp OR 'arteria mesenterica superior' OR 'mesenteric artery'/exp OR 'mesenteric artery' OR 'mesenteric arteries'/exp OR 'mesenteric arteries' OR 'coeliac trunk'/exp OR 'coeliac trunk' OR 'truncus coeliacus'/exp OR 'truncus coeliacus' OR 'visceral artery'/exp OR 'visceral artery' OR 'visceral arteries' OR 'celiac artery'/exp OR 'celiac artery' OR 'celiac arteries' 38,839
#2. 'dissection'/exp OR dissection 229,672
#3. #1 AND #2 3,592
#4. #1 AND #2 AND [1995-2020]/py 3,416
#5. #4 AND ('Article'/it OR 'Article in Press'/it OR 'Editorial'/it OR 'Letter'/it OR 'Review'/it) 2,248

Cochrane Library, 2020-02-17

#1 MeSH descriptor: [Mesenteric Artery, Superior] explode all trees 64
#2 MeSH descriptor: [Celiac Artery] explode all trees 20
#3 ("superior mesenteric artery" OR "arteria mesenterica superior" OR "mesenteric artery" OR "mesenteric arteries" OR "coeliac trunk" OR "truncus coeliacus" OR "visceral artery" OR "visceral arteries" OR "celiac artery" OR “celiac arteries”):ti,ab,kw (Word variations have been searched) 538
#4 #1 OR #2 OR #3 538
#5 (dissection):ti,ab,kw (Word variations have been searched) 8690
#6 MeSH descriptor: [Dissection] explode all trees 252
#7 #5 OR #6 8703
#8 #4 AND #7 80

Number of hits in all databases	3,789
Number of hits after deduplication in EndNote	2,531

Extra search using the MESH term ”aneurysm, dissecting” resulted in an additional 74 articles. Eight of these were relevant, but all were case reports and were excluded from the study.
